# The Accuracy and Reliability of Crowdsource Annotations of Digital Retinal Images

**DOI:** 10.1167/tvst.5.5.6

**Published:** 2016-09-21

**Authors:** Danny Mitry, Kris Zutis, Baljean Dhillon, Tunde Peto, Shabina Hayat, Kay-Tee Khaw, James E. Morgan, Wendy Moncur, Emanuele Trucco, Paul J. Foster, 1 for the UK Biobank Eye and Vision Consortium

**Affiliations:** 1 NIHR Biomedical Research Centre at Moorfields Eye Hospital & UCL Institute of Ophthalmology, London, UK; 2 VAMPIRE project, School of Science and Engineering, University of Dundee, Dundee, UK; 3 Centre for Clinical Brain Sciences, University of Edinburgh and Princess Alexandra Eye Pavilion, Edinburgh, UK; 4 Department of Public Health and Primary Care, University of Cambridge Strangeways Research Laboratory, Worts Causeway, Cambridge, UK; 5 Department of Clinical Gerontology, Addenbrookes Hospital, University of Cambridge, Cambridge, UK; 6 School of Optometry and Vision Sciences, Cardiff University, Cardiff, UK; 7 Duncan of Jordanstone College of Arts and Design, University of Dundee, Dundee, UK

**Keywords:** retina, image analysis, crowdsourcing

## Abstract

**Purpose:**

Crowdsourcing is based on outsourcing computationally intensive tasks to numerous individuals in the online community who have no formal training. Our aim was to develop a novel online tool designed to facilitate large-scale annotation of digital retinal images, and to assess the accuracy of crowdsource grading using this tool, comparing it to expert classification.

**Methods:**

We used 100 retinal fundus photograph images with predetermined disease criteria selected by two experts from a large cohort study. The Amazon Mechanical Turk Web platform was used to drive traffic to our site so anonymous workers could perform a classification and annotation task of the fundus photographs in our dataset after a short training exercise. Three groups were assessed: masters only, nonmasters only and nonmasters with compulsory training. We calculated the sensitivity, specificity, and area under the curve (AUC) of receiver operating characteristic (ROC) plots for all classifications compared to expert grading, and used the Dice coefficient and consensus threshold to assess annotation accuracy.

**Results:**

In total, we received 5389 annotations for 84 images (excluding 16 training images) in 2 weeks. A specificity and sensitivity of 71% (95% confidence interval [CI], 69%–74%) and 87% (95% CI, 86%–88%) was achieved for all classifications. The AUC in this study for all classifications combined was 0.93 (95% CI, 0.91–0.96). For image annotation, a maximal Dice coefficient (∼0.6) was achieved with a consensus threshold of 0.25.

**Conclusions:**

This study supports the hypothesis that annotation of abnormalities in retinal images by ophthalmologically naive individuals is comparable to expert annotation. The highest AUC and agreement with expert annotation was achieved in the nonmasters with compulsory training group.

**Translational Relevance:**

The use of crowdsourcing as a technique for retinal image analysis may be comparable to expert graders and has the potential to deliver timely, accurate, and cost-effective image analysis.

## Introduction

Telemedicine is an effective way to deliver high quality medical care to virtually any location, and aims to overcome the issue of access to specialist services.^1^ The UK National Diabetic Retinopathy Screening programs are examples of the successful use of telemedicine in ophthalmology. Diabetic retinopathy is a leading cause of visual impairment in working age adults. Appropriately validated digital imaging technology has been shown to be a sensitive and effective screening tool to identify patients with diabetic retinopathy for referral for ophthalmic evaluation and management.^2^ The National Screening Committee currently recommends annual screening for all persons with diabetes over the age of 12 years (available in the public domain at http://legacy.screening.nhs.uk/diabeticretinopathy). With the number of individuals with diabetes expected to reach 366 million globally by 2030, and the prevalence of retinopathy ranging between 30% and 56% in diabetics, screening services are likely to continue to be a significant cost in future healthcare provision.^3,4^ In the United Kingdom, the estimated per-individual cost for annual screening is £25 to £35.^5^ Based on the estimated adult prevalence of diabetes in the United Kingdom in 2013,^6^ this translates to an annual cost of £102 to £135 million for diabetic retinopathy screening.

Timely, accurate and repeatable retinal image analysis and interpretation is a critical component of ophthalmic telemedicine programs. However, retinal image analysis is labor-intensive, involving highly trained graders interpreting multiple images through complex protocols. Computerized, semiautomated image analysis techniques have been developed that may be able to reduce the workload and screening costs; however, these methods are not widely used at present.^7^ As telemedicine continues to expand, more cost-effective techniques will need to be developed to manage the high volume of images expected, particularly in the context of public health screening.

Crowdsourcing is the process of outsourcing computationally intensive tasks to many untrained individuals. It involves simplifying a large, complex task into smaller parts, which can be completed by a group of untrained individuals in the general public.^8^ Its use in medical image analysis remains uncommon, but early studies have shown that this technique can provide a combination of accuracy and cost effectiveness that has the potential to significantly advance medical image analysis delivery.^9–12^ Crowdsourcing has been used successfully to decipher complex protein folding structures with an online game (available in the public domain at http://foldit.wikia.com/wiki/Foldit_Wiki) and solve complex medical cases (available in the public domain at https://www.crowdmed.com/). In addition, it has been used in video-based assessment of surgical technique, with nonmasters users performing as well as experts.^13^ Knowledge workers (KWs) also were used in the classification of polyps on CT scans which demonstrated an area under the curve (AUC) of 0.845 ± 0.045 compared to expert grading with minimal online training.^10^ We demonstrated that the sensitivity of the crowdsourcing interface to detect severe retinal abnormalities from fundus images can be ≥96% and between 61% and 79% for mild abnormalities.^9^

Using retinal images derived from the EPIC-Norfolk Eye Study,^14^ our aim was to develop a modifiable webpage to interact with the online distributing platform (Amazon Mechanical Turk) to allow for more complex task development and user training, and to create a tool to visually engage the participant, allowing direct image annotation.

## Methods

### Ethics Statement

The European Prospective Investigation of Cancer (EPIC)–Norfolk third health examination (3HC) was reviewed and approved by the East Norfolk and Waverney NHS Research Governance Committee (2005EC07L) and the Norfolk Research Ethics Committee (05/Q0101/ 191). Local research and development approval was obtained through Moorfield's Eye Hospital, London (FOSP1018). The research was conducted in accordance with the principles of the Declaration of Helsinki. All participants gave written, informed consent.

The European Prospective Investigation of Cancer is a pan-European study that started in 1989 with the primary aim of investigating the relationship between diet and cancer risk.^14^ The 3HC was carried out between 2006 and 2011 with the objective of investigating various physical, cognitive and ocular characteristics of 8623 participants then aged 48 to 91 years. A detailed eye examination including mydriatic fundus photography was attempted on all participants in the 3HC using a Topcon TRC NW6S camera (Topcon Corporation, Tokyo, Japan). A single image of the macular region and optic disc (field 2 of the modified Airlie House classification) was taken of each eye.^15^

Two clinicians (DM, PF) and two senior retinal photography graders selected, by consensus, a series of 100 retinal images from the EPIC Norfolk 3HC. The images chosen had a mixture of retinal pathology comprising retinal vascular occlusions, diabetic retinopathy, and age-related maculopathy. We selected images with predetermined criteria: severely abnormal images (*N* = 10) were determined as having grossly abnormal findings, comprising a central macular scar or peripheral hemorrhages in more than 2 quadrants. Mildly abnormal images (*N* = 60) were designated if there was macular pigmentation or drusen or peripheral hemorrhages in 2 quadrants or less. Normal images (*N* = 30) had no discernible pathology. Appendix 1 demonstrates example images. All images were anonymized and uploaded onto an ftp site for the study duration to allow remote access. “Healthy” images are those classified as normal, “nonhealthy” images are those classified as mildly or severely abnormal, which could comprise yellow/black spots and/or hemorrhage.

### Website Design

#### Technology

The website back-end was created in PHP 5.3, in conjunction with a MySQL 5.5 database to manage serving images, storing annotation data, and tracking user annotation sessions. The front end relies heavily on the Javascript jQuery library for managing user interaction and data capture. The server is hosted in Apache 2.2 running on CentOS 6.4. (available in the public domain at www.retinalannotations.com)

#### Motivation and Layout

The site's main goal was to create an easy-to-use and intuitive interface, with users giving annotations via minimal mouse input. We opted to split the annotation into two stages; initial image class classification (identify image as healthy or nonhealthy), and a second “click and drag” rectangle-based input for localizing abnormal regions. Simple rectangle localization is significantly faster than segmenting the detailed contour of the region. The annotators also were assigned a unique code, which could be used to retrieve previous session data and results when visiting the site at a later date, so that the whole image set could be annotated conveniently in multiple sessions. Live feedback was given after each image was annotated illustrating the type of annotation performed by the majority of other users.

The site was linked to Amazon Mechanical Turk (AMT) which was used to drive traffic to the site. The annotation task was listed in the public repository for workers to view. Participants were directed to the retinalannotations.com website, to carry out annotations, and finally report their unique user code in the AMT interface.

#### Usability

A usability study was carried out by an external expert in the field (WM). The resulting report was used to refine the site format and functionality to conform to best practice standards for accessibility and usability.

#### Amazon Mechanical Turk (AMT)

We used the AMT web platform to drive traffic to our site, providing anonymous workers access to perform classification tasks on the fundus photographs in our dataset. Amazon Mechanical Turk employs KWs, who are unselected, registered Amazon users who carry out simple tasks. Each KW receives a small monetary reward from the requester for each task that is completed to a satisfactory standard (5 cents per image annotated in our case). Amazon keeps a record of the performance of each KW and designates a “masters qualification” to a small number of individuals who perform exceptionally well in a large number of tasks.

#### Training and Study Design

Before starting an annotation task, all users had (optional) access to a training area, where they were asked to classify and annotate 16 practice images. Feedback on the accuracy of the user's practice annotations was given in the form of a visual overlay prepared by a senior retinal image grader.

The remaining 84 retinal images were published as one human intelligence task (HIT), in AMT terminology. Each KW had to complete all 84 classification tasks to secure remuneration. Each KW could complete the task only once. No demographic data were collected on KWs completing the task and no nationality restrictions were placed. Based on previous estimations of repeated task accuracy in distributed human intelligence tasks, we requested 20 KW classifications per image.^10^ After using unrestricted KWs, we conducted the study two more times: a second time using only those designated with the Amazon Masters qualification, and a third time using unrestricted KWs but specifying that they complete the training module with a minimum of 80% accuracy. The monetary reward remained unchanged for all tests.

#### Analysis

Using the expert graded images as the “ground truth,” we calculated the sensitivity and specificity for differentiating healthy versus nonhealthy, and compared the performance of KW's designated masters to nonmasters. The AUC of the receiver operating characteristic (ROC) curve, was calculated as a global index of diagnostic accuracy. For annotation accuracy, each rectangle from the annotations was converted into a binary mask. We used the Dice coefficient to assess the overlap or consensus between pairs of binary masks (the thresholded average annotation mask [0.25] versus the expert annotation). The Dice coefficient quantifies the overlap of two image regions, and is defined as the size of intersection versus the size of union. The Dice coefficient measure takes into account the size and location of the annotated lesions. The Dice coefficient ranges from 0 (no overlap) to 1 (complete congruence) with values >0.6 considered to denote a substantial level of agreement.^16^ Correlation plots were used to examine the relationship in area of annotation (not the location) between clinician grader and crowdsourced annotators. Matlab 2013b (MathWorks, Natick, MA) was used for all statistical analysis.

## Results

In total, we received 5389 classifications for 84 images (excluding the 16 training images). Due to incomplete submissions from some KWs there was a small difference in the number of KWs in each group. A total of 2113 classifications from the unrestricted nonmasters was received in 48 hours (25 unique KWs), 1596 classifications from the masters only qualification group were received in 13 days (19 unique KWs), and 1680 classifications from the nonmasters with compulsory training group were received in 11 days (20 unique KWs). Table 1 illustrates the sensitivity and specificity of the classifications. The sensitivity and specificity were calculated based on the individual responses of all users who completed the task. The proportion of images correctly classified by the majority (>50%) of KWs was between 90% and 100% for all groups for healthy and nonhealthy images with the exception of nonmasters with compulsory training, where the majority of KWs correctly classified only 62.5% of normal images. Table 2 illustrates the percentage of correct (healthy and nonhealthy) classifications across all groups.

**Table 1:**
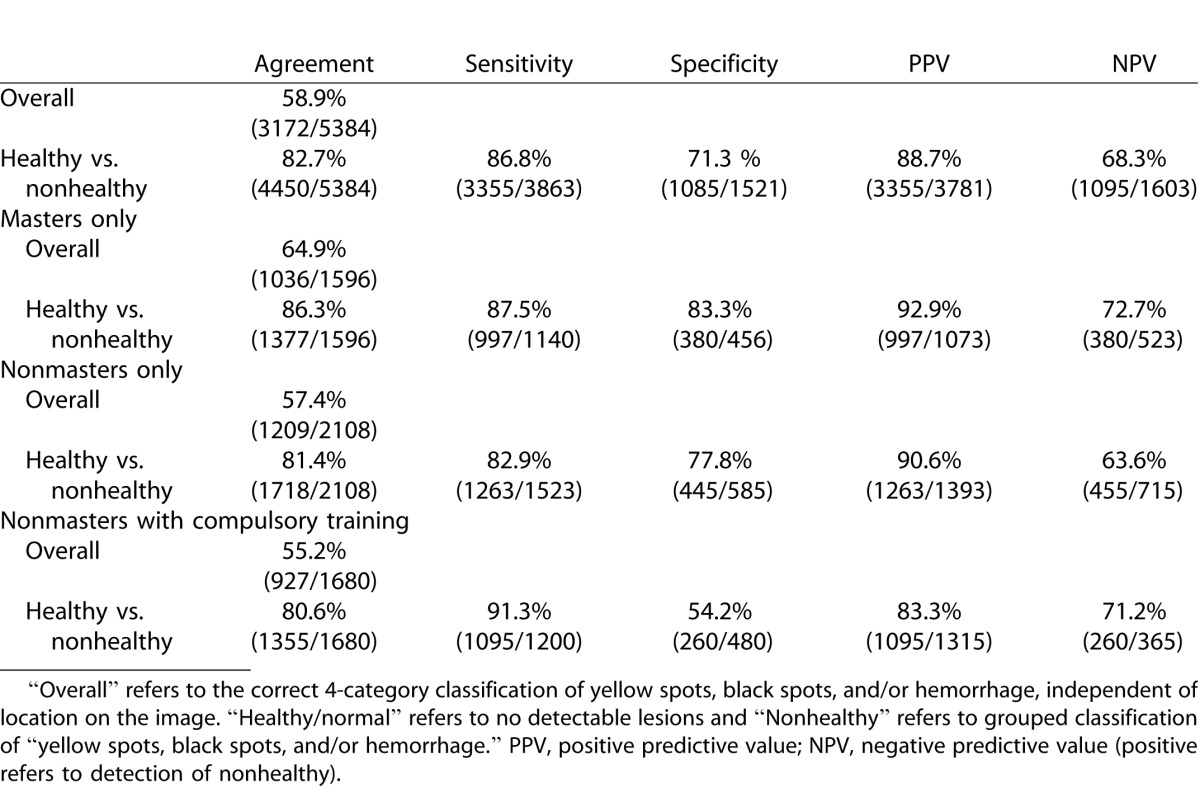
The Specificity and Sensitivity Overall and by Masters and Nonmasters for the Correct Detection of Healthy/Nonhealthy Images

**Table 2:**
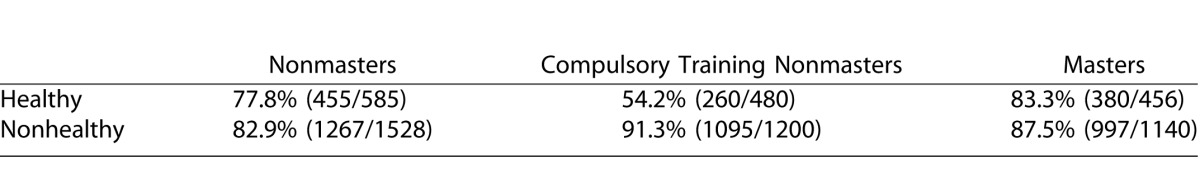
The Percentage of Correct Image Class Classifications across for Healthy and Nonhealthy Images across All Groups

### Annotation Accuracy

**Figure 1:**
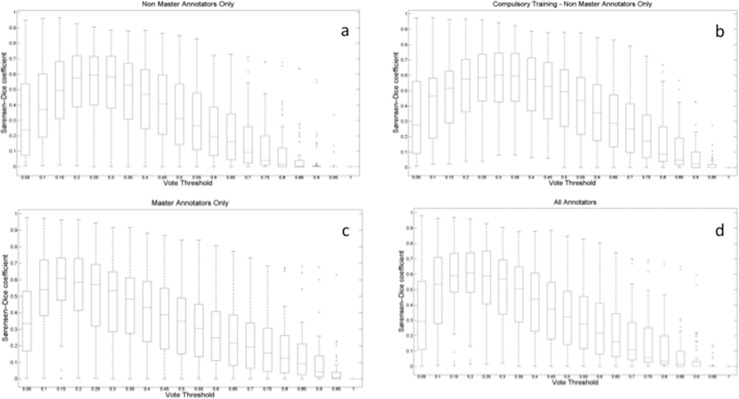
Plots the dice coefficient (clinician/KW pixel annotation congruence) against the vote threshold (proportion of votes). The dice coefficient (*y*-axis) increases as the proportion of votes (*x*-axis) increases, achieving an optimal value when approximately a 25% consensus is achieved to mark a pixel as an abnormal lesion. (a) Nonmasters only annotators. At the 0.25 threshold, the median (95% CI) Dice coefficient for nonmasters only was 0.59 (0.54–0.65). (b) Nonmasters compulsory training annotators. At the 0.25 threshold, the median (95% CI) Dice coefficient for nonmasters only was 0.59 (0.53–0.64). (c) Masters only annotators. At the 0.25 threshold, the median (95% CI) Dice coefficient for Masters only was 0.57 (0.51–0.63). (d) All annotators. At the 0.25 threshold, the median (95% CI) Dice coefficient for all annotators was 0.59 (0.53–0.65).

Before analysis, the 24 normal images where no annotation was appropriate (normal) were excluded. For each remaining image (*N* = 60), the user's rectangles in the database were converted into a binary mask, and all user masks then were averaged to create an average mask across all the users for that image. Thresholds (0.15, 0.2, 0.25) then were applied to turn the average image into a binary mask. Figures 1a to d illustrates the median Dice coefficient, the consensus threshold for all annotators, and the masters and nonmasters groups, suggesting an optimal threshold of 0.25. The raw overlapping annotation pixels (omitted for reasons of space) can be viewed at http://retinalannotations.com/publications/AnnotationTimelapseRaw.mp4 and projected over an example image: (available in the public domain at http://retinalannotations.com/publications/AnnotationTimelapseOverlay.mp4).

### Correlation

**Figure 2:**
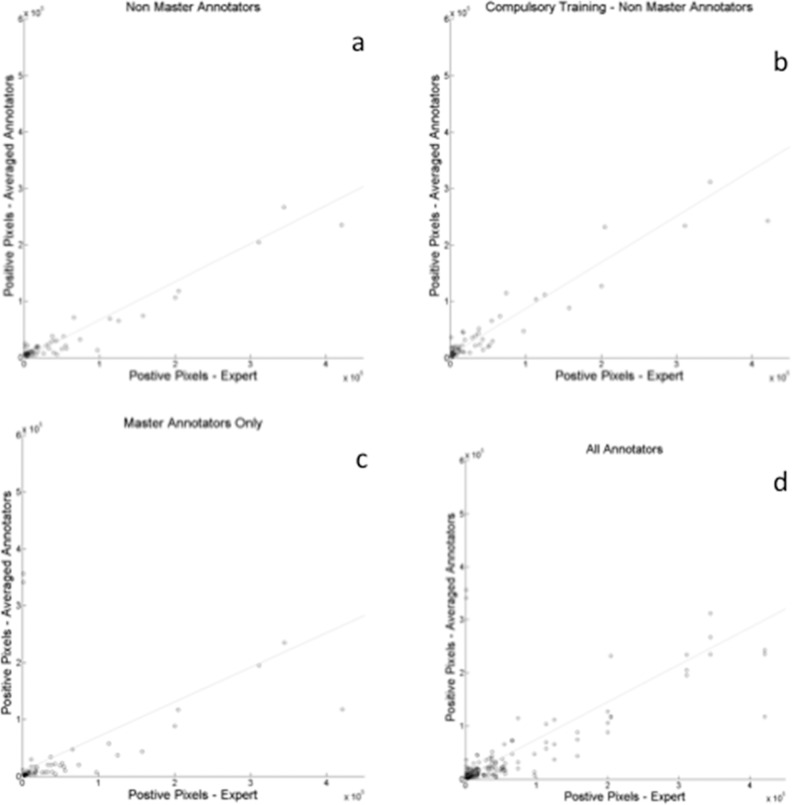
Correlation plots illustrating the relationship between averaged user image annotation and expert annotation for (a) nonmasters only annotators, (b) nonmaster compulsory training annotators, (c) masters-only annotators, and (d) all annotators.

Figure 2 represents the correlation between expert and averaged user annotation of positive pixel designation using the web annotation tool. To construct this plot, for each nonhealthy image (1–60), we created an “average” annotator image (by adding all annotator images, and dividing by number of annotators for that image) and threshold it at the optimal threshold (0.25). We then counted the number of “positive” (i.e., pixels marked as lesions) pixels in the thresholded image to plot on the *y*-axis, and plot the number of “positive” pixels in the corresponding expert annotation of that image on the *x*-axis. This correlation plot does not report accuracy of the location of the annotations, but rather reports the “area” of pixels marked as lesioned by the annotators compared to the expert. For all annotators, the correlation coefficient was 0.87. For masters KW's only users it was 0.70 and for nonmasters only (compulsory and noncompulsory training) it was unchanged at 0.97.

### ROC Curves

**Figure 3:**
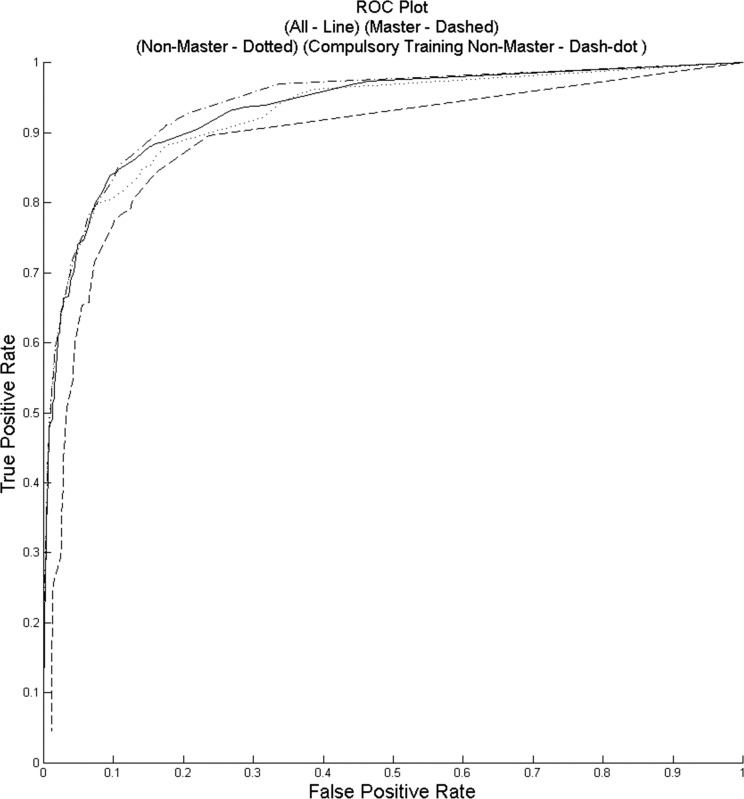
Illustrates to AUC for nonmasters only annotators (*dotted*), nonmaster compulsory training annotators (*dash*-*dot*), masters-only annotators (*dashed*), and all annotators (*line*).

Figure 3 illustrates the ROC curves for each of the groups versus expert grading in the classification of healthy and nonhealthy images. The AUC for nonmasters only was 0.93 (95% CI, 0.90–0.96), for nonmasters compulsory training was 0.94 (95% CI, 0.91–0.97), for masters only was 0.89 (95% CI, 0.87–0.91) and for all annotators was 0.93 (95% CI, 0.91–0.96).

## Discussion

Crowdsourcing represents an effective and rapid method of annotating retinal images with scalability that has potentially far-reaching implications in public health and, in particular, in relation to screening programs. Crowdsourcing has been used as a novel method for medical image classification and its utility and accuracy in diagnosis has been explored recently.^9–11,17^ To our knowledge, this is the first study to demonstrate that untrained user consensus annotation of abnormalities of clinical relevance in retinal images is comparable to clinician annotation.

Overall, a specificity and sensitivity of 71% and 87% was achieved for all classifications. We demonstrated that a maximal Dice coefficient (∼0.6, implying a high level of agreement) can be achieved with a consensus threshold of only 25% for this task. Masters-only graders demonstrated a higher accuracy than nonmasters graders (64% vs. 55%–57%). A higher sensitivity and lower specificity were seen in the nonmasters compulsory training group compared to the nonmasters group alone, suggesting a greater tendency towards false-positives with additional training. The reason for this is unclear but may relate to interpretation bias introduced during the compulsory training and different levels of understanding of the training exercise.^18^ The strongest correlation was seen between the nonmasters users (with or without compulsory training) and clinician annotation. Masters only users showed greater deviation when compared to clinician graders. The reasons behind this are uncertain, but may relate to how Master users are designated by Amazon.

One of the limitations of this study is that the annotation tool only allowed one or multiple rectangles to be drawn, which may affect the users' ability to capture the region of interest if, for example, the region was irregular in shape or size. While more simple tasks can be carried out effectively, increasing task complexity can result in reduced performance. Using images of diabetic retinopathy, Brady et al.^19^ suggested a reduction in correct classifications (*N* = 230) from 81% to 50% when the classification options were increased from binary to four possible categories. Maintaining classification accuracy in complex tasks with high clinical variability remains a challenge and optimal parameters for training, incentive rewards, and maintaining user interest remain uncertain. However, using the collective intelligence of the crowd to interpret the subtlety of retinal images provides a theoretical flexibility that may not be realised in automated segmentation. By using multiple repetitions for each task and a majority vote, outliers likely to be spurious can be effectively excluded.^20^ Furthermore, the potential for analysis of interannotator variability among the expert graders in this study is limited, which may influence the reported findings.

The AUC in this study for all classifications combined was 0.93 (95% CI, 0.91–0.96). This is similar to large-scale validated automated diabetic systems versus expert grading 0.937 (95% CI, 0.916–0.959).^21–23^ Similarly, our previous study (using the same images) used a simple classification proforma without training images or annotation and achieved an AUC range of 0.70 (95% CI, −0.68–0.78) across all trials.^9^ The improved AUC in this present study suggests that with minimal additional training the diagnostic accuracy can be improved significantly.

Behavioral research has suggested that crowdsourcing has the potential to be a revolutionary tool for conducting experiments, allowing large experiments to be run cost-effectively in a number of hours. Crowdsourcing also may target a more representative general population that varies in age, education, ethnicity, and geographic location.^24^ The optimal design for a crowdsource-based experiment is likely to vary with the complexity of the task involved. However, similar to our work, other researchers have identified that accuracy is dependent on testing the users' comprehension of the task before participation. Financial incentive is not directly related to accuracy but generally results in a higher rate of participation.^10,13,24^ Recent research also suggests that a high dropout rate, resulting from perhaps boredom with the task, or a sense of inadequate financial reward, is a factor that may adversely affect accuracy.^24^

In conclusion, crowdsourcing has significant potential as a cost-effective and accurate technique with applicability as a screening tool in retinal image analysis. Our study confirmed that accuracy and reproducibility are comparable to clinician graders, and can be achieved at a fraction of the cost of trained graders. Larger, appropriately incentivized studies with novel techniques of training nonexperts are likely to be important in establishing user comprehension and maintaining user interest in more complex annotation tasks.

## Supplementary Material

Supplement 1
