# Supplementary material for: The Accuracy and Reliability of Crowdsource Annotations of Digital Retinal Images
Source: Transl Vis Sci Technol. 2016 Sep 21;5(5):6. doi: 10.1167/tvst.5.5.6 (PMC5032847; doi:10.1167/tvst.5.5.6)
Supplement: Supplement 1 [file i2164-2591-5-5-6-s01.pdf]

Example images

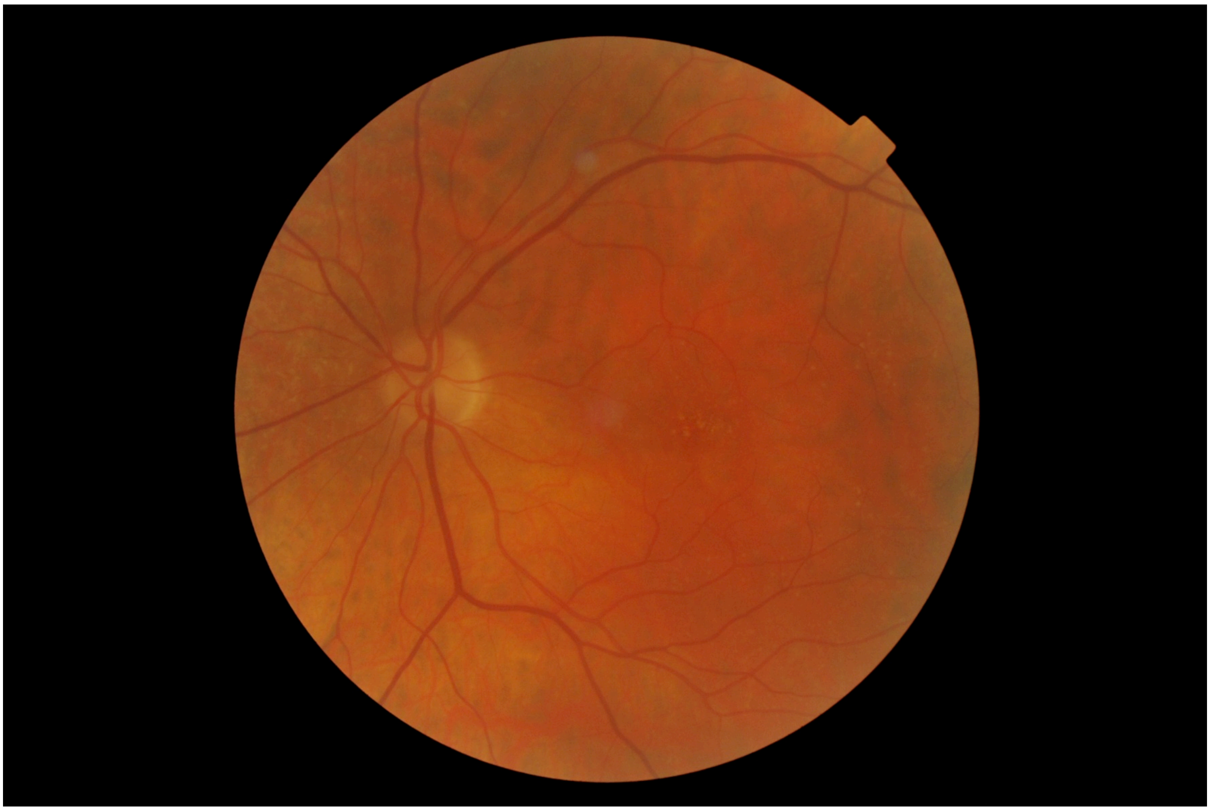

Figure 1: An example of mild abnormality

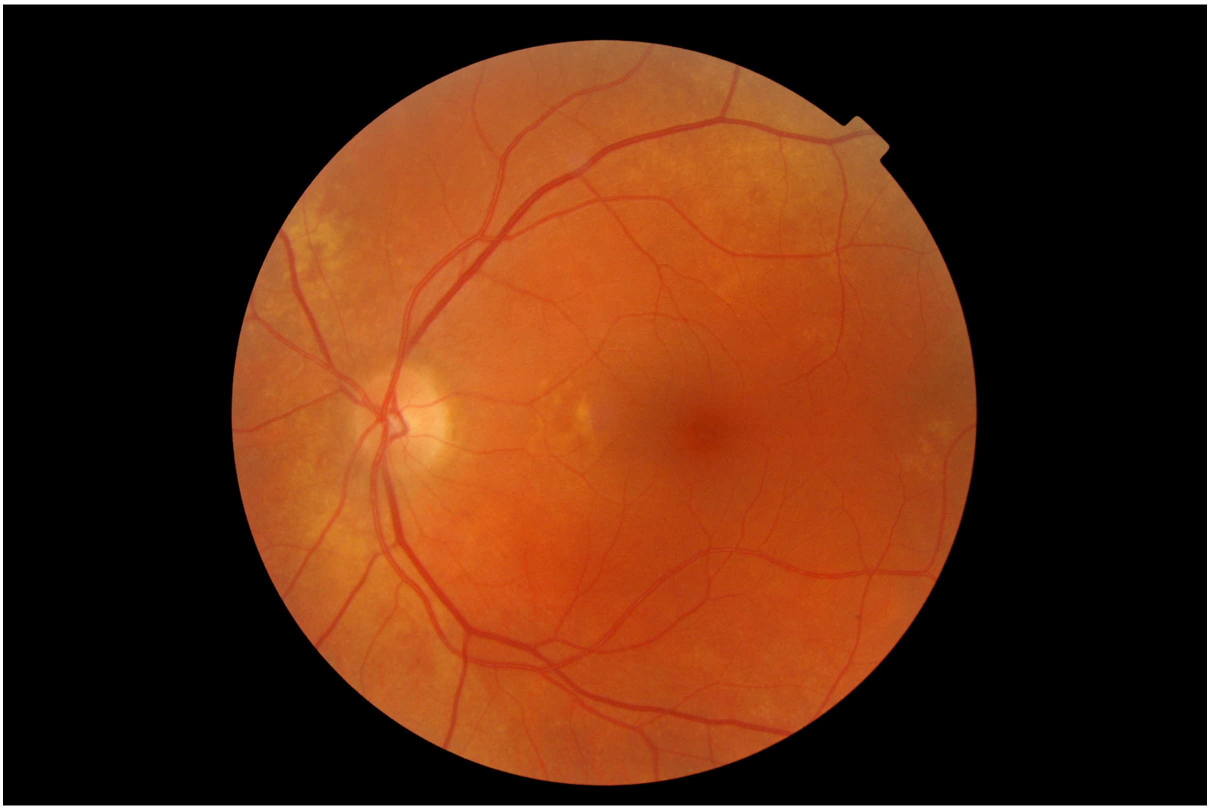

Figure 2: An example of mild abnormality

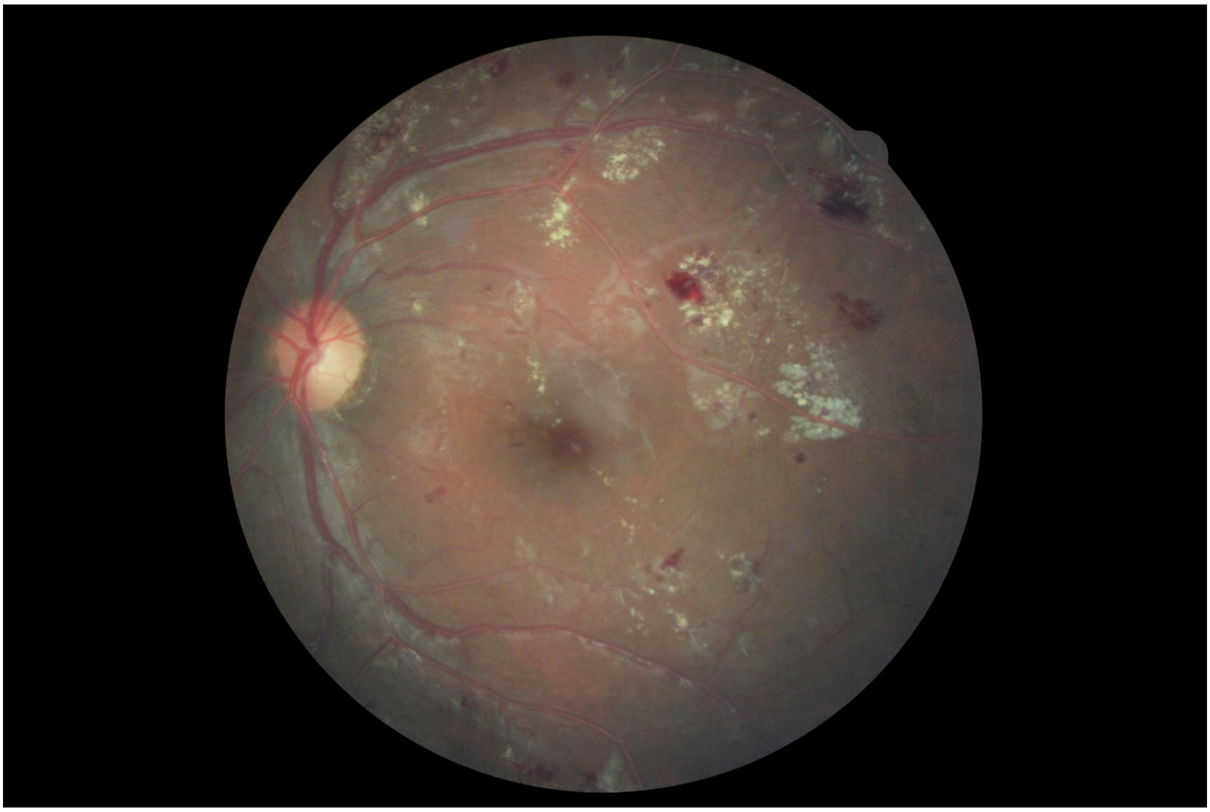

Figure 3: An example of severe abnormality
